# Economic Outcomes of Patients Receiving Antiretroviral Therapy for HIV/AIDS in South Africa Are Sustained through Three Years on Treatment

**DOI:** 10.1371/journal.pone.0012731

**Published:** 2010-09-14

**Authors:** Sydney Rosen, Bruce Larson, Alana Brennan, Lawrence Long, Matthew Fox, Constance Mongwenyana, Mpefe Ketlhapile, Ian Sanne

**Affiliations:** 1 Boston University Center for Global Health and Development, Boston, Massachusetts, United States of America; 2 Health Economics and Epidemiology Research Office, Wits Health Consortium, Faculty of Health Sciences, University of the Witwatersrand, Johannesburg, South Africa; 3 Clinical HIV Research Unit, Faculty of Health Sciences, University of the Witwatersrand, Johannesburg, South Africa; Albert Einstein College of Medicine, United States of America

## Abstract

**Background:**

Although the medical outcomes of antiretroviral therapy (ART) for HIV/AIDS are well described, less is known about how ART affects patients' economic activities and quality of life, especially after the first year on ART. We assessed symptom prevalence, general health, ability to perform normal activities, and employment status among adult antiretroviral therapy patients in South Africa over three full years following ART initiation.

**Methodology/Principal Findings:**

A cohort of 855 adult pre-ART patients and patients on ART for <6 months was enrolled and interviewed an average of 4.4 times each during routine clinic visits for up to three years after treatment initiation using an instrument designed for the study. The probability of pain in the previous week fell from 74% before ART initiation to 32% after three years on ART, fatigue from 66% to 12%, nausea from 28% to 4%, and skin problems from 55% to 10%. The probability of not feeling well physically yesterday fell from 46% to 23%. Before starting ART, 39% of subjects reported not being able to perform their normal activities sometime during the previous week; after three years, this proportion fell to 10%. Employment rose from 27% to 42% of the cohort. Improvement in all outcomes was sustained over 3 years and for some outcomes increased in the second and third year.

**Conclusions/Significance:**

Improvements in adult ART patients' symptom prevalence, general health, ability to perform normal activities, and employment status were large and were sustained through the first three years on treatment. These results suggest that some of the positive economic and social externalities anticipated as a result of large-scale treatment provision, such as increases in workforce participation and productivity and the ability of patients to carry on normal lives, may indeed be accruing.

## Introduction

By late 2008, some 2.9 million people in sub-Saharan Africa were receiving antiretroviral therapy for HIV/AIDS[1]. A large body of literature confirms the success of AIDS treatment programs in reducing viral loads and restoring the immune systems of adult patients in sub-Saharan Africa[2]–[6]. Long-term survival rates remain high despite some concerns about long-term retention of patients in treatment programs[7].

Alongside these studies of biomedical outcomes of treatment is a small body of literature investigating the impacts of ART on patients' economic activities, quality of life, and other non-biomedical outcomes during the first year on treatment. These studies, which have recently been reviewed[8], have consistently reported rapid and relatively large improvements in quality of life and/or work attendance during the first year on ART among adult patients in Africa. After 12 months on ART, for example, large proportions of patients participating in a prospective cohort study in Cape Town, South Africa reported improvements in all domains assessed, which included self-care, usual activities, pain/discomfort, and anxiety/depression[9]. Similarly, after 12 months on ART, patients enrolled in a prospective cohort in Uganda showed significantly better scores in all domains studied, including role function, general health perceptions, mental health, physical health, and depression[10].

What has been missing so far is evidence about the longer-term sustainability of these improvements beyond the initial year on treatment. Sustained improvements in economic and quality of life outcomes are likely to support long-term adherence and to mitigate some of the negative economic and social consequences of untreated HIV/AIDS in high-prevalence countries. In this paper, we report results of a longitudinal study of South African ART patients who have been followed for up to three years after starting treatment. Outcomes analyzed include self-reported ability to perform normal daily activities, prevalence of symptoms, and employment status in the first 1080 days after ART initiation.

## Methods

### Ethics statement

The study was approved by the Institutional Review Board of the Boston University Medical Campus and the Human Research Ethics Committee (Medical) of the University of the Witwatersrand. Written informed consent was provided by all study subjects.

### Study sites

The study was conducted at three ART clinics in South Africa. The cohort and study methods have been described previously[11]–[13]. The first site, the Themba Lethu Clinic of Helen Joseph Hospital in Gauteng Province, is a large, urban, public referral hospital which had 9,600 adult patients on ART as of September 2009. The other two sites are nongovernmental clinics. The Witkoppen Health and Welfare Centre is a full service, primary care clinic serving informal peri-urban settlements in Gauteng Province. ACTS Clinic is a dedicated HIV/AIDS clinic in a rural setting in Mpumalanga Province. As of September 2009, these two sites had approximately 2,700 and 2,400 adult patients on ART, respectively.

All three sites followed the 2004 South African national guidelines for providing HIV/AIDS care and treatment. Under these guidelines, ART was initiated once a patient's CD4 count fell to less than 200 cells/mm^3^ or WHO Stage IV disease was diagnosed, though many patients first presented at the study clinics with CD4 counts far below 200 Most patients were initiated on the national first-line regimen of stavudine, lamivudine, and efavirenz or nevirapine. Patients not yet eligible for treatment were monitored and received counseling and vitamin supplements.

### Sample selection and data collection

At each site, study subjects were identified each day from patients present at the clinic, either waiting in a queue or attending an adherence or wellness class or support group. We selected subjects from these groups (queue, class, or support group) using nth-name sampling and invited them to participate in the study. Adult patients who were HIV-positive but not yet medically eligible for ART or who had initiated ART less than six months before recruitment were eligible for enrollment. Those who provided written informed consent were enrolled in the cohort and administered a baseline questionnaire by a study interviewer. Enrollment took place between June 2005 and June 2006.

Follow-up questionnaires were then administered as often as possible when subjects returned for consultations or to pick up medications. Because clinic visits were often unplanned and not all subjects had time to participate in an interview at every visit, interviews occurred at only a fraction of total visits and at irregular intervals, as is discussed further below. In addition, subjects' medical records were reviewed and information collected about date of ART initiation, dates of clinic visits, CD4 counts, and other indicators. For this analysis, data were censored on September 30, 2009.

### Outcome variables

The questionnaire was designed for this study and focused on subjects' self-reported health conditions and engagement in economic activities. For this analysis, nine specific outcomes are investigated. Four are related to specific symptoms: pain or headache, nausea, tiredness or fatigue, and skin rash or other skin problems. Because these symptoms affect a person's ability to perform normal activities, these outcomes have economic as well as quality-of-life implications. Two outcomes are related to general health condition and asked whether the subject did not feel well physically at least part of the day yesterday or whether the subject felt sad, depressed, or stressed at least part of the day yesterday. Finally, three outcomes pertained to normal activities and employment status. Inability to perform normal activities was defined as reporting being unable to perform one's normal activities for at least part of a day in the previous five-day work week (Monday-Friday) due to physical or mental health. When subjects reported any inability to perform normal activities, the number of days to which this pertained in the same five-day period was elicited. The employment status variable was defined as holding a full time or part time wage or salary position, including domestic service but excluding informal sector piecework activities. Although many of our outcomes overlap with definitions in the World Health Organization's International Classification of Functioning, Disability and Health (ICF)[14], for this study multiple ICF classifications were collapsed into a few more broadly-defined outcomes.

### Statistical analysis

Information from the nine outcomes was organized into 8 dichotomous variables (1 = yes, 0 = no) and one continuous variable (days unable to perform normal activities in previous work week, between 0 and 5). For each interview for each study subject who initiated ART prior to June 2009, the interview date was compared to the date the subject initiated therapy to calculate the number of days on ART at each interview. The date of ART initiation was extracted from the subject's clinical records. Time on ART, from 90 days before initiation ART to 1080 days after, was stratified into thirteen 90-day increments. In recognition that changes in wellbeing may be more rapid immediately after initiating treatment, the first 90-day period was further subdivided into days 0–30 and 31–90 on ART, for a total of 14 separate time-on-ART dummy variables.

A logistic regression framework was used to estimate the probability (absolute risk) that a subject reported yes for each dichotomous outcome, with data pooled across the three study sites. Linear regression was used for the number of days of unable to perform normal activities. A population-averaged model for all outcomes was used to account for multiple observations on a single subject (using xtlogit and xtreg in STATA 10 with the pa option). In each model, variables included three age categories (18–29, 40–49, 50+), sex (male = 1, female = 0), and 13 time-on-ART dummy variables. The reference age category variable was 30–39 years of age and the reference time-on-ART variable was the initial 30 days on ART (days 0–30). Other than for the employment status outcome, significant site effects were not found, so site was not included as a variable in the models. For the employment status outcome, the site-specific estimates are discussed in the results section.

Using the logistic regression results, we computed the estimated probability for each outcome for the reference case representing the largest age/sex group in the sample (female, aged 30–39) during the first month on ART (0–30 days on ART). We then estimated marginal/partial effects for each covariate in the logistic model (other age categories, male instead of female, and the 13 time-on-ART dummy variables) using the mfx post-estimation command in STATA 10. Odds ratios from the logistic regression model are not reported but available from the authors. Finally, we performed Wald tests for linear hypotheses about the parameters of the logistic regression model to investigate if the changes observed after 360 days on ART were sustained over the next two years (using the post-estimation test command in STATA 10.1).

## Results

### Cohort recruitment and baseline characteristics

**Figure 1:**
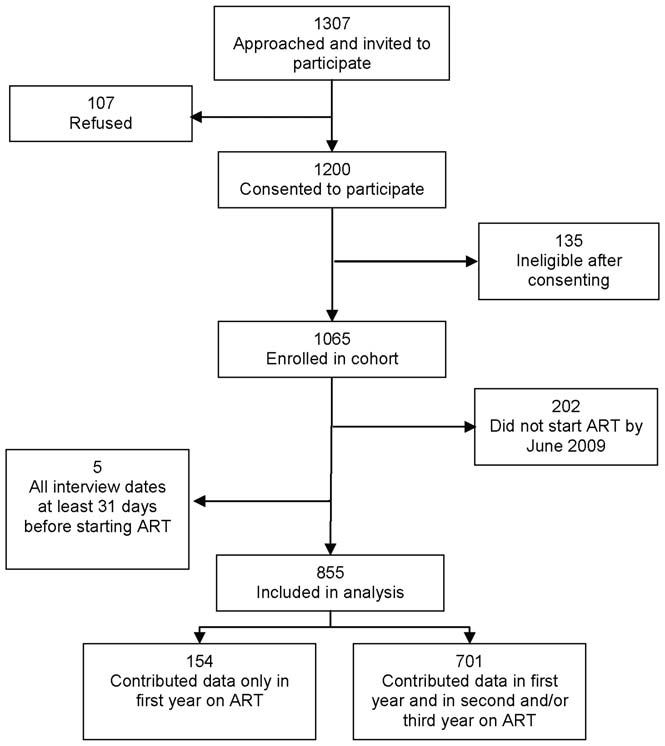
Study subject recruitment and inclusion for analysis.

The cohort was enrolled between July 1, 2005 and June 30, 2006. Study recruitment and the construction of the analytic cohort are shown in Figure 1. Characteristics of the cohort at baseline have been described previously[12], [13]. At baseline, the cohort included a total of 1,065 subjects, of whom 449 had not yet started ART and 616 had been on ART for less than 6 months. At the time of censoring for data analysis, 57% of the original cohort of 1,065 subjects were still attending the study clinics and had been interviewed at least once within the previous 12 months; 8% were still attending the study clinics but had not been interviewed within the previous 12 months (study loss to follow up); 22% were no longer attending the study clinics for unknown reasons (program loss to follow up); 7% had transferred to a different treatment facility; and 5% had died.

In this paper, we report data collected between the baseline interview and September 30, 2009. During this period of observation, 855 subjects initiated or were already on ART and completed at least one interview after treatment initiation. The remaining 210 study subjects had not yet started ART by their final interview within this period or were not interviewed between 90 days before ART initiation and 1080 after and are not included in this analysis. For the 855 subjects included, the average number of interviews per subject was 4.4. Interviews were conducted primarily during visits for purposes of medical consultation (59%) or medication pickup (38%). The median [IQR] interval between interviews was 170 [115–266] days.

Table 1 summarizes demographic characteristics of the cohort at baseline. Most study subjects were women, which reflects the preponderance of female patients at the site. Almost all were economically active, either outside the home in formal (25%) or informal (19%) employment or inside the home (26%) performing housework or child care. Most of the rest (24%) were seeking employment.

**Table 1:** Characteristics of cohort at baseline.

Variable	Value
N	855
Female (%)	662 (77%)
Age at enrollment (%)	
18–29 years	242 (28%)
30–39 years	402 (47%)
40–49 years	160 (19%)
>49 years	51 (6%)
Median CD4 count closest to treatment initiation (cells/mm^3^) [IQR]	105 [37–161]
Primary activity when feeling well enough to perform normal activities, at study enrollment (n (%))	
Employed in formal sector	217 (25%)
Work in informal sector or self-employed	166 (19%)
Unemployed, seeking work	204 (24%)
Housework or family care (unpaid)	223 (26%)
Other (studying, retired, leisure, service, missing)	45 (5%)

**Figure 2:**
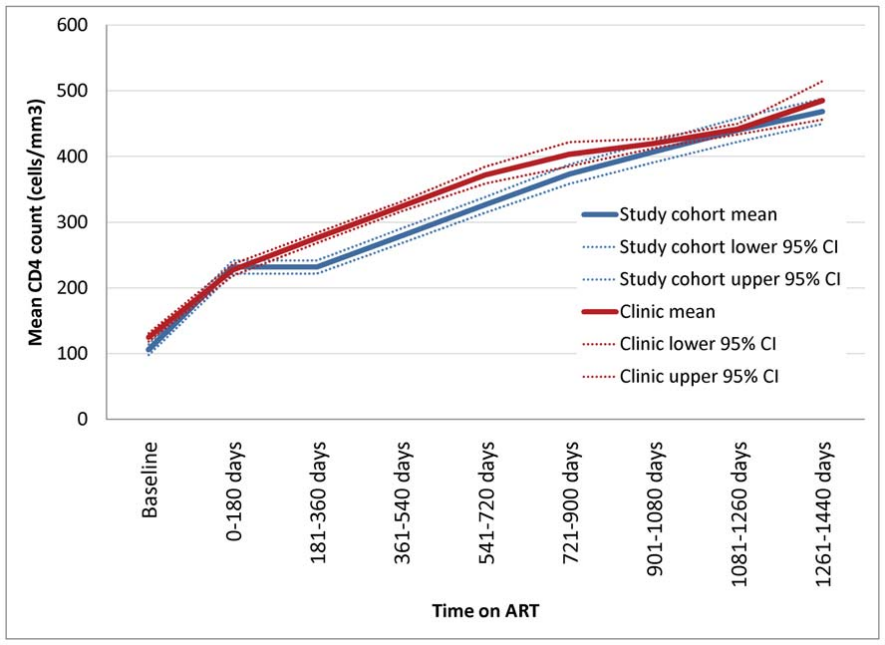
Mean CD4 cell counts of study cohort and clinic population by time on ART.

Most subjects initiated treatment at low CD4 counts (median 105 cells/mm^3^). As Figure 2 illustrates, mean CD4 counts among study subjects improved steadily as duration on treatment increased. Figure 2 also indicates that the mean CD4 counts of all patients initiated on ART at our largest study site, Themba Lethu Clinic in Johannesburg, are similar to those of the study cohort. Confidence intervals of the two groups overlap in the first six months and after two years on treatment; between months 6 and 24, the difference between them is small, with the study cohort's mean slightly lower than that of the clinic population overall.

### Symptoms – fatigue, pain, nausea, and skin problems

Table 2 and Table 3 report the probability that subjects were unable to perform their normal activities for at least part of a day in the previous 7-day week as a result of each of four symptoms (fatigue, pain, nausea, and skin problems). For each symptom, the tables first report the estimated probability for the reference case (female, aged 30–39, on ART for 0–30 days). For example, the estimated probability of reporting pain for the reference case was 0.543 (54.3%). The following rows in the table report the marginal effect or change in the estimated probability for each separate variable included in the regression model, the probability for the time interval, and the 95% confidence interval and associated p-value for each marginal effect. For pain, the marginal effect for the variable sex (male) is −0.104, leading to an estimated probability of reporting pain of 0.543–0.104 = 43.9% for men, aged 30–39, and on ART between 0 and 30 days.

**Table 2:** Reference probability and marginal effects of experiencing pain or fatigue during the previous week (logistic regression, population averaged model).

Variable	Marginal effect	Probability	p-value	95% confidence interval for marginal effect
*Outcome: Pain last week*				
Reference case estimated probability = 0.543*				
sex (male)	−0.104	43.9%	0.000	[−0.156,−0.052]
18–29 years old	−0.053	49.0%	0.038	[−0.103,−0.003]
40–49 years old	0.082	62.5%	0.003	[0.028,0.136]
50+ years old	0.066	60.9%	0.123	[−0.018,0.150]
−90 to −1 days	0.201	74.4%	0.000	[0.109,0.293]
0 to 30 days (reference)	*	54.3%	*	*
31–90 days	−0.038	50.5%	0.397	[−0.126,0.050]
91–180 days	−0.052	49.1%	0.193	[−0.131,0.026]
181–270 days	−0.024	51.9%	0.589	[−0.110,0.062]
271–360 days	−0.076	46.7%	0.087	[−0.162,0.011]
361–450 days	−0.098	44.5%	0.026	[−0.184,−0.012]
451–540 days	−0.133	41.0%	0.003	[−0.221,−0.045]
541–630 days	−0.135	40.8%	0.003	[−0.224,−0.045]
631–720 days	−0.178^a^	36.5%	0.000	[−0.264,−0.093]
721–810 days	−0.278	26.5%	0.000	[−0.357,−0.199]
811–900 days	−0.189	35.4%	0.000	[−0.267,−0.110]
901–990 days	−0.25	29.3%	0.000	[−0.327,−0.173]
991–1080 days	−0.220^a^	32.3%	0.000	[−0.297,−0.142]
*Outcome: Fatigue last week*				
*Reference case estimated probability = 0.498*				
sex (male)	−0.068	43.0%	0.021	[−0.126,−0.01]
18–29 years old	0.004	50.2%	0.894	[−0.051,0.059]
40–49 years old	0.035	53.3%	0.283	[−0.028,0.098]
50+ years old	0.054	55.2%	0.265	[−0.041,0.15]
−90 to −1 days	0.158	65.6%	0.002	[0.06,0.256]
0 to 30 days (reference)	*	49.8%	*	*
31–90 days	−0.023	47.5%	0.614	[−0.111,0.066]
91–180 days	−0.17	32.8%	0.000	[−0.247,−0.092]
181–270 days	−0.171	32.7%	0.000	[−0.254,−0.087]
271–360 days	−0.226	27.2%	0.000	[−0.309,−0.144]
361–450 days	−0.241	25.7%	0.000	[−0.323,−0.160]
451–540 days	−0.249	24.9%	0.000	[−0.332,−0.166]
541–630 days	−0.275	22.3%	0.000	[−0.358,−0.191]
631–720 days	−0.292^b^	20.6%	0.000	[−0.371,−0.212]
721–810 days	−0.341	15.7%	0.000	[−0.416,−0.266]
811–900 days	−0.328	17.0%	0.000	[−0.402,−0.254]
901–990 days	−0.372	12.6%	0.000	[−0.444,−0.300]
991–1080 days	−0.374^a^	12.4%	0.000	[−0.445,−0.302]

*Reference case is a female subject age 30–39, 1–30 days on ART.

a Indicates p-value ≤0.05 for Wald test that marginal effect equals marginal effect for 271–360 days on ART.

b Indicates p-value >0.05 for Wald test that marginal effect equals marginal effect for 271–360 days on ART.

**Table 3:** Reference probability and marginal effects of experiencing nausea or skin problems during the previous week (logistic regression, population averaged model).

Variable	Marginal effect	Probability	p-value	95% confidence interval for marginal effect
*Outcome: Nausea last week*				
*Reference case estimated probability = 0.231*				
sex (male)	−0.018	21.3%	0.488	[−0.069,0.033]
18–29 years old	0.018	24.9%	0.484	[−0.033,0.070]
40–49 years old	0.028	25.9%	0.374	[−0.033,0.089]
50+ years old	−0.014	21.7%	0.759	[−0.105,0.076]
−90 to −1 days	0.048	27.9%	0.286	[−0.040,0.137]
0 to 30 days (reference)	*	23.1%	*	*
31–90 days	−0.03	20.1%	0.423	[−0.103,0.043]
91–180 days	−0.107	12.4%	0.001	[−0.169,−0.044]
181–270 days	−0.099	13.2%	0.004	[−0.166,−0.031]
271–360 days	−0.097	13.4%	0.005	[−0.164,−0.029]
361–450 days	−0.109	12.2%	0.001	[−0.176,−0.043]
451–540 days	−0.136	9.5%	0.000	[−0.202,−0.071]
541–630 days	−0.154	7.7%	0.000	[−0.219,−0.089]
631–720 days	−0.168^a^	6.3%	0.000	[−0.231,−0.106]
721–810 days	−0.185	4.6%	0.000	[−0.245,−0.124]
811–900 days	−0.181	5.0%	0.000	[−0.241,−0.122]
901–990 days	−0.184	4.7%	0.000	[−0.244,−0.125]
991–1080 days	−0.190^a^	4.1%	0.000	[−0.250,−0.131]
*Outcome: Skin problems last week*				
*Reference case estimated probability = 0.507*				
sex (male)	−0.036	47.1%	0.230	[−0.096,0.023]
18–29 years old	−0.01	49.7%	0.725	[−0.066,0.046]
40–49 years old	−0.016	49.1%	0.631	[−0.082,0.050]
50+ years old	−0.039	46.8%	0.466	[−0.142,0.065]
–90 to −1 days	0.041	54.8%	0.425	[−0.060,0.142]
0 to 30 days (reference)	*	50.7%	*	*
31–90 days	0.001	50.8%	0.979	[−0.088,0.090]
91–180 days	−0.122	38.5%	0.002	[−0.201,−0.043]
181–270 days	−0.143	36.4%	0.001	[−0.229,−0.058]
271–360 days	−0.173	33.4%	0.000	[−0.259,−0.087]
361–450 days	−0.295	21.2%	0.000	[−0.376,−0.214]
451–540 days	−0.325	18.2%	0.000	[−0.406,−0.244]
541–630 days	−0.35	15.7%	0.000	[−0.431,−0.269]
631–720 days	−0.405^a^	10.2%	0.000	[−0.480,−0.330]
721–810 days	−0.41	9.7%	0.000	[−0.484,−0.337]
811–900 days	−0.42	8.7%	0.000	[−0.491,−0.348]
901–990 days	−0.438	6.9%	0.000	[−0.509,−0.367]
991–1080 days	−0.408^a^	9.9%	0.000	[−0.479,−0.336]

*Reference case is a female subject age 30–39, 1–30 days on ART.

a Indicates p-value ≤0.05 for Wald test that marginal effect equals marginal effect for 271–360 days on ART.

**Figure 3:**
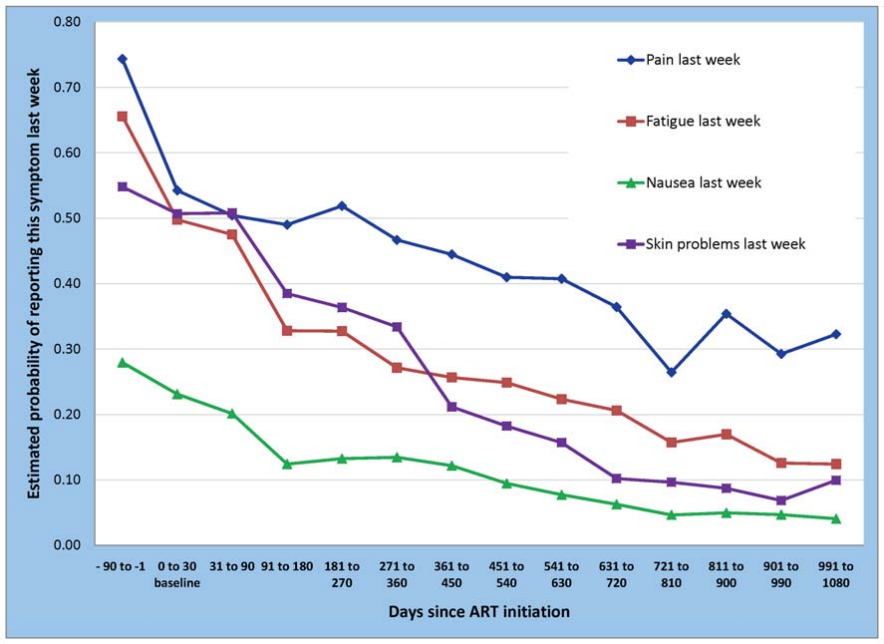
Probability of experiencing symptoms last week by time on ART.

The probabilities of each symptom for the reference case (women, aged 30–39) over the 14 time categories are illustrated in Figure 3. Declines in the probability of reporting physical symptoms in the past week were significant (p-value ≤0.05) and large for all four symptoms investigated (fatigue, pain, nausea, skin problems). Improvements were sustained and or become larger over the entire period of observation. The estimated probability of pain for the reference case was 0.54. Prior to initiating ART (days -90 to -1), the estimated probability of pain was substantially higher, 0.74. Once ART was started, the declines estimated were not significantly different from zero up to 360 days (i.e. the probability of reporting pain in the previous week did not fall during the initial year on therapy). By 720 days on ART, however, the probability of experiencing pain in the previous week had fallen to 0.36, a large and significant decline of 33% from baseline. After three years, the probability had fallen further to 0.32, or 41% below baseline. The probability after two years on ART was estimated to be significantly lower than both the baseline and the one year values. The estimated improvement in year two was sustained through year three.

Fatigue, nausea, and skin problems showed a similar pattern of ongoing improvement, with symptom probabilities falling by 80–85% between 0–90 days before ART initiation and three years after. Men were estimated to experience significantly less pain and fatigue than women, but no sex differences were estimated in the probability of nausea or skin problems.

### General health on day preceding interview

**Figure 4:**
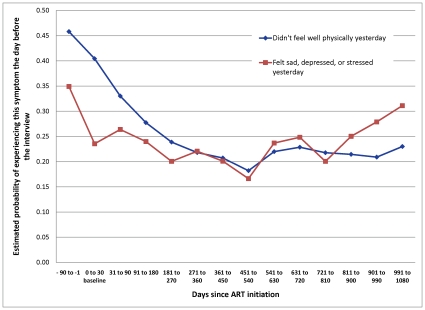
Probability of not feeling well on day prior to interview by time on ART.

Subjects were also asked two general questions about how they felt on the day preceding the interview (did not fell well physically yesterday for part or all of the day; felt sad, depressed, or stressed yesterday for part or all of the day). Table 4 presents the logistic regression model results, estimated marginal effects, and probabilities for these two outcomes, and Figure 4 illustrates the estimated probabilities for each outcome over time on ART.

**Table 4:** Reference probability and marginal effects of feeling unwell on day before interview (logistic regression, population averaged model).

Variable	Marginal effect	Probability	p-value	95% confidence interval for marginal effect
*Outcome: Felt unwell physically yesterday (part or all of the day)*				
*Reference case estimated probability = 0.404*				
sex (male)	−0.055	34.9%	0.030	[−0.104,−0.005]
18–29 years old	−0.033	37.1%	0.186	[−0.082,0.016]
40–49 years old	0.086	49.0%	0.003	[0.030,0.142]
50+ years old	0.081	48.5%	0.062	[−0.004,0.166]
–90 to −1 days	0.054	45.8%	0.299	[−0.048,0.155]
0 to 30 days (reference)	*	40.4%	*	*
31–90 days	−0.074	33.0%	0.092	[−0.160,0.012]
91–180 days	−0.127	27.7%	0.001	[−0.204,−0.050]
181–270 days	−0.166	23.8%	0.000	[−0.247,−0.084]
271–360 days	−0.186	21.8%	0.000	[−0.267,−0.106]
361–450 days	−0.197	20.7%	0.000	[−0.277,−0.117]
451–540 days	−0.222	18.2%	0.000	[−0.302,−0.142]
541–630 days	−0.184	22.0%	0.000	[−0.268,−0.101]
631–720 days	−0.176^b^	22.8%	0.000	[−0.257,−0.094]
721–810 days	−0.187	21.7%	0.000	[−0.265,−0.109]
811–900 days	−0.19	21.4%	0.000	[−0.265,−0.114]
901–990 days	−0.195	20.9%	0.000	[−0.270,−0.120]
991–1080 days	−0.174^b^	23.0%	0.000	[−0.250,−0.099]
*Outcome: Felt sad, depressed, or stressed yesterday (all or part of the day)*				
*Reference case estimated probability = 0.236*				
sex (male)	−0.018	21.8%	0.334	[−0.056,0.019]
18–29 years old	−0.011	22.5%	0.565	[−0.048,0.026]
40−49 years old	0.041	27.7%	0.078	[−0.005,0.087]
50+ years old	0.064	30.0%	0.086	[−0.009,0.136]
−90 to −1 days	0.114	35.0%	0.017	[0.021,0.207]
0 to 30 days (reference)	*	23.6%	*	*
31–90 days	0.028	26.4%	0.47	[−0.049,0.105]
91–180 days	0.004	24.0%	0.899	[−0.063,0.072]
181–270 days	−0.035	20.1%	0.339	[−0.107,0.037]
271–360 days	−0.015	22.1%	0.692	[−0.088,0.059]
361–450 days	−0.035	20.1%	0.347	[−0.107,0.038]
451–540 days	−0.069	16.7%	0.057	[−0.141,0.002]
541–630 days	0.001	23.7%	0.973	[−0.076,0.079]
631–720 days	0.013^b^	24.9%	0.737	[−0.062,0.088]
721–810 days	−0.035	20.1%	0.321	[−0.104,0.034]
811–900 days	0.015	25.1%	0.678	[−0.055,0.084]
901–990 days	0.043	27.9%	0.224	[−0.026,0.113]
991–1080 days	0.076^a^	31.2%	0.035	[0.005,0.146]

*Reference case is a female subject age 30–39, 0–30 days on ART.

a Indicates p-value ≤0.05 for Wald test that marginal effect equals marginal effect for 271–360 days on ART.

b Indicates p-value >0.05 for Wald test that marginal effect equals marginal effect for 271–360 days on ART.

The estimated probability that a subject reported feeling unwell physically yesterday fell sharply over the first year on ART, from 0.40 to 0.22, an improvement that was sustained over the next two years on therapy. The estimated probability of feeling sad, depressed, or stressed yesterday did not improve, however. While the probability of this outcome at baseline (0–30 days) is significantly lower than in the 90 days prior to treatment initiation, there are no further estimated improvements that are sustained for more than two time periods.

### Normal activities and employment

**Figure 5:**
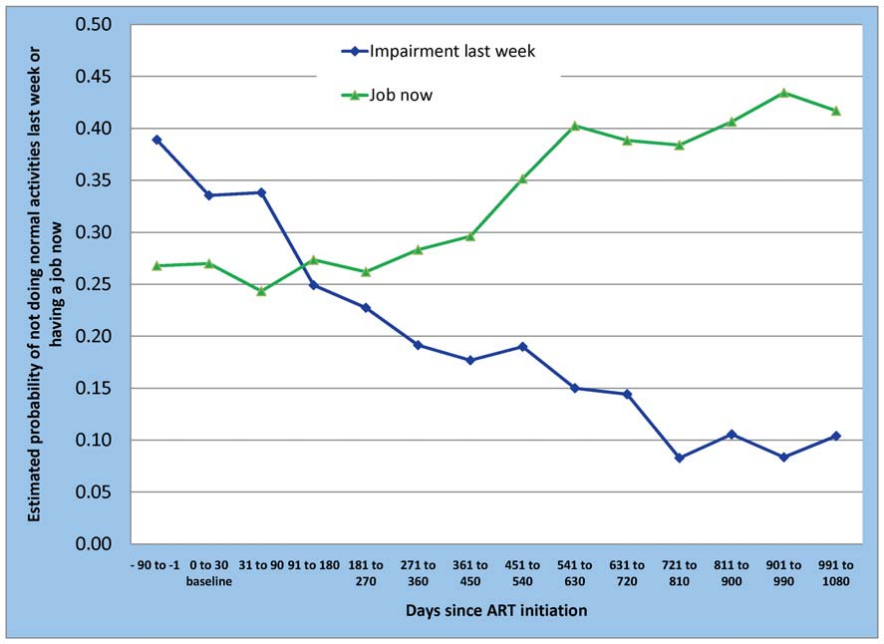
Probability of being unable to perform normal activities last week and of employment by time on ART.

Subjects were also asked about their ability to perform the primary normal daily activities reported in Table 1 during the previous five-day work week. If any inability was reported, then the number of days was elicited. Table 5 reports results for both of these outcomes, and the estimated probability of reporting at least part of a day in the previous week is illustrated in Figure 5. For the reference case, this probability fell from 0.50 in the period preceding the start of ART to approximately 0.21 after 1 year on ART, 0.15 after two years on ART, and 0.11 by the end of three years. There were no significant differences in this outcome between males and females. Older patients were slightly more likely to report this outcome.

**Table 5:** Reference probability, marginal effects, and duration of inability to perform normal activities in previous work week.

Variable	Marginal effect	Probability or number of days	p-value	95% confidence interval for marginal effect
*Outcome: Unable to perform normal activities in previous week because of problems with physical or mental health^c^*				
*Reference case estimated probability = 0.356*				
sex (male)	−0.039	31.7%	0.164	[−0.095,0.016]
18–29 years old	−0.02	33.6%	0.466	[−0.075,0.034]
40–49 years old	0.07	42.6%	0.034	[0.005,0.135]
50+ years old	0.033	38.9%	0.517	[−0.067,0.133]
–90 to −1 days	0.140	49.6%	0.005	[0.041,0.240]
0 to 30 days (reference)	*	35.6%	*	*
31–90 days	0.003	35.9%	0.949	[−0.082,0.087]
91–180 days	−0.09	26.6%	0.017	[−0.163,−0.016]
181–270 days	−0.112	24.4%	0.005	[−0.191,−0.034]
271–360 days	−0.15	20.6%	0.000	[−0.227,−0.073]
361–450 days	−0.166	19.0%	0.000	[−0.242,−0.089]
451–540 days	−0.152	20.4%	0.000	[−0.230,−0.073]
541–630 days	−0.194	16.2%	0.000	[−0.271,−0.117]
631–720 days	−0.200^b^	15.6%	0.000	[−0.275,−0.126]
721–810 days	−0.266	9.0%	0.000	[−0.335,−0.197]
811–900 days	−0.241	11.5%	0.000	[–0.311,−0.172]
901–990 days	−0.265	9.1%	0.000	[−0.333,−0.197]
991–1080 days	−0.243^a^	11.3%	0.000	[−0.312,−0.175]
*Outcome: Number of days unable to perform normal activities in previous week because of problems with physical or mental health if any inability in previous week^d^*				
*Reference case estimated number of days = 3.036*				
sex (male)	−0.001	3.303	0.994	[−0.330,0.327]
18–29 years old	−0.111	3.193	0.487	[−0.426,0.203]
40–49 years old	0.054	3.358	0.759	[−0.288,0.396]
50+ years old	0.092	3.396	0.735	[−0.441,0.625]
−90 to −1 days	0.543	3.847	0.046	[0.009,1.077]
0 to 30 days (reference)	*	3.304	*	*
31–90 days	0.089	3.393	0.734	[−0.425,0.603]
91–180 days	0.09	3.394	0.719	[−0.401,0.581]
181–270 days	0.567	3.871	0.046	[0.010,1.124]
271–360 days	0.745	4.049	0.015	[0.144,1.346]
361–450 days	0.437	3.741	0.161	[−0.174,1.048]
451–540 days	0.36	3.664	0.252	[−0.255,0.976]
541–630 days	0.16	3.464	0.649	[−0.527,0.847]
631–720 days	−0.056^a^	3.248	0.869	[−0.719,0.608]
721–810 days	0.122	3.426	0.751	[−0.635,0.880]
811–900 days	−0.174	3.130	0.600	[−0.822,0.475]
901–990 days	0.05	3.354	0.889	[−0.654,0.754]
991–1080 days	−0.759^a^	2.545	0.020	[−1.400,−0.118]

*Reference case is a female subject age 30–39, 0–30 days on ART.

a Indicates p-value ≤0.05 for Wald test that marginal effect equals marginal effect for 271–360 days on ART.

b Indicates p-value >0.05 for Wald test that marginal effect equals marginal effect for 271–360 days on ART.

c Logistic regression, population averaged model.

d Linear regression, population averaged model; marginal effect  =  linear regression coefficient.

Although the estimated probability of any inability to perform normal activities in the previous week fell dramatically over the first year on ART, the estimated average duration of inability for those who reported any inability remained relatively long regardless of time on ART. Conditional on any inability, the estimated average number of days unable to perform normal activities for the reference case is 3.03 days in the previous five working days, or roughly 60% of the work week.

Finally, the results for employment status are reported in Table 6 and Figure 5. The estimated probability of having a job began to increase after one and a half years on ART and remained significantly higher, in the neighborhood of 45% of the cohort, for the rest of the follow up period. The relatively long delay in seeing a change in this indicator, rather than a large improvement during the first year as with most other outcomes, may reflect the time required to find employment once one becomes capable of working. There is no indication that official unemployment in South Africa fell during the study period; official unemployment was 24.2% in March 2005[15], just before study recruitment began, and 24.5% in September 2009[16], when data were censored. The increase in employment observed for our cohort over time on ART thus cannot be explained by improvements in the broader labor market.

**Table 6:** Reference probability and marginal effects of being employed at time of interview (logistic regression, population averaged model).

Variable	Marginal effect	Probability	p-value	95% confidence interval for marginal effect
*Outcome: Currently have a job*				
*Reference case estimated probability = 0.308*				
sex (male)	0.062	37.0%	0.070	[−0.005,0.129]
18–29 years old	−0.038	27.0%	0.214	[−0.098,0.022]
40–49 years old	0.032	34.0%	0.390	[−0.041,0.105]
50+ years old	0.023	33.1%	0.687	[−0.091,0.138]
−90 to −1 days	−0.002	30.6%	0.942	[−0.067,0.062]
0 to 30 days (reference)	*	30.8%	*	*
31–90 days	−0.029	27.9%	0.313	[−0.085,0.027]
91–180 days	0.004	31.2%	0.880	[−0.046,0.054]
181–270 days	−0.009	29.9%	0.750	[−0.062,0.045]
271–360 days	0.014	32.2%	0.609	[−0.040,0.069]
361–450 days	0.028	33.6%	0.312	[−0.026,0.083]
451–540 days	0.087	39.5%	0.003	[0.029,0.144]
541–630 days	0.14	44.8%	0.000	[0.081,0.199]
631–720 days	0.125^a^	43.3%	0.000	[0.069,0.181]
721–810 days	0.121	42.9%	0.000	[0.067,0.174]
811–900 days	0.144	45.2%	0.000	[0.092,0.196]
901–990 days	0.172	48.0%	0.000	[0.120,0.224]
991–1080 days	0.155^a^	46.3%	0.000	[0.103,0.206]

*Reference case is a female subject age 30–39, 0–30 days on ART.

a Indicates p-value ≤0.05 for Wald test that marginal effect equals marginal effect for 271–360 days on ART.

b Indicates p-value >0.05 for Wald test that marginal effect equals marginal effect for 271–360 days on ART.

Unlike for other outcomes, employment status did vary by study site. If Themba Lethu Clinic, the large urban public hospital in Johannesburg, is used as the reference case, subjects enrolled at the Witkoppen Health and Welfare Centre, which serves informal settlements on the edge of Johannesburg, were 8% more likely to report being employed, while those at the ACTS Clinic, a rural site in Mpumalanga Province, were 7% less likely to report being employed. For all sites, the basic trend over time on ART remained essentially the same as shown in Figure 5.

## Discussion

In a cohort of 855 adult patients on ART at three treatment facilities in South Africa, indicators of well-being and economic activity improved significantly within one year of treatment initiation and either continued to improve or remained stable for a full three years after starting treatment. For those patients who remain on treatment, ART appears to offer long-term gains in ability to perform normal activities, symptom prevalence, and employment opportunities. The findings of this study suggest that some of the positive economic and social externalities anticipated as a result of large-scale treatment provision, such as increases in workforce participation and productivity and the ability of patients to carry on normal lives, may indeed be accruing.

By the end of the three-year observation period, the probability of pain, fatigue, nausea, and skin problems reported by study subjects had fallen by 57–85%. While pain and fatigue at three years remained relatively common (reported by 32% and 12% of subjects, respectively), these should be interpreted cautiously, as some residual level of pain and fatigue is likely to be reported even by a healthy, HIV-negative population. The probability of reporting any inability to perform normal activities for reasons of health during the preceding five-day work week fell to a stable level of 10% of the cohort. For this core indicator of wellbeing, a decline from almost 40% when initiating therapy to 10% represents an important gain.

The study reported here has several limitations. First, it is an on-treatment analysis only. The enrollment refusal rate was low (8%), but post-enrollment loss to follow up from the cohort was high, for a number of reasons. The most common reason was that the patient had stopped attending the study clinic, due to program loss to follow up (22%), transfer to another site (7%), or death (5%). Study loss to follow up—patients who had made visits in the 12 months before data censoring but not been interviewed—was 8%. Discontinuation of treatment may well be correlated with our outcomes of interest, if patients who felt worse or better than average were more likely to drop out of treatment or avoid being interviewed. Our results should therefore be interpreted as conditional on staying alive and on treatment at the same site. As indicated by the CD4 counts shown in Figure 2, however, the study cohort does not appear to have substantially better or worse immunological outcomes than the broader population of ART patients at the largest study site.

A second potential source of bias is the timing of interviews, which took place on days when patients presented voluntarily to the study clinics. It is likely that some visits during which interviews were conducted were due to patient condition, and not for routine monitoring or drug pickup. The study sites' records do not allow us to distinguish clearly between routine and non-routine medical consultations. If many interviews took place during visits for patient condition, then our results could understate the average benefits of treatment later in the study. Since study staff did not approach patients who appeared visibly ill and unable to participate in an interview, and because most interviews were conducted during routine visits, we believe that this potential bias is modest. On the other hand, decisions not to interview patients who appeared visibly ill, which were infrequent but did occur, could lead to a slight overestimate of the benefits of treatment.

In addition, we frequently had long and irregular gaps between interviews, resulting in a very unbalanced data set and differing numbers of observations among subjects followed for the same overall length of time. This resulted largely from the irregularity of routine patient visits to the study clinics and the difficulty of locating and interviewing patients who present at unscheduled times. Finally, as with any study that relies on self-reported interview data, recall error is likely. We deliberately chose very short recall periods (≤ 7 days) to minimize this risk, but we could not eliminate entirely.

Our results are roughly consistent with those of several studies that have reported only to 12 months after treatment initiation. In the study in Cape Town, South Africa, for example, the proportion of patients reporting no problems with usual activities rose from 76% at baseline to 94% at 12 months.[9] Similarly, in Uganda the prevalence of functional impairment declined from 50% to 7% after 12 months on ART.[17] In the only study we are aware of that reports to two years, the probability of Kenyan agricultural workers on ART experiencing bodily pain often in the previous week fell from 24.3% at treatment initiation to 9.9% after two years, while the probability of fatigue declined from 40.5% to 14.8%.[18] While the two-year probabilities in this study were lower than in ours, the magnitude of the declines—roughly 60%—is similar. What we were able to show is that these improvements endure through at least the third year on treatment.

We conclude that patients who remain on ART in South Africa experience large improvements in their ability to perform normal activities, symptom prevalence, and employment potential and that these improvements are sustained and for some outcomes continue to increase over the first three years on ART. While we have no way to know how our study subjects compare with HIV-negative South Africans of comparable socioeconomic status, the relatively low prevalence of self-reported inability to perform normal activities and of symptoms, and the relatively high employment rate after three years, suggest that ART patients, assuming that they remain on treatment, are able to lead relatively normal lives.
